# Supplementary material for: Pathogenic Leptospira species identified in dogs and cats during neutering in Thailand
Source: PLoS Negl Trop Dis. 2026 Feb 4;20(2):e0013421. doi: 10.1371/journal.pntd.0013421 (PMC12871963; doi:10.1371/journal.pntd.0013421)
Supplement: S1 File — (DOCX) [file pntd.0013421.s001.docx]

**S1 File Identification of *Leptospira* species**

**Partial 16S rRNA sequence amplification and analysis**

The nested PCR assay targeting partial 16S rRNA sequences of P1 and P2 was conducted as described previously, with modifications [1]. The first 25 μl PCR reaction was modified as follows: master mix containing 1.25 units of Taq DNA polymerase (iNtRON Biotechnology Inc, Gyeonggi-do, South Korea), 1х MgCl_2_ free PCR buffer, 2.5 mM of MgCl_2_, 0.2 mM of dNTP (iNtRON Biotechnology Inc, Gyeonggi-do, South Korea), 5 μl of 5 M of Betaine (Bio Basic USA Inc., New York, US) and each reaction consist of 0.5 pmol each of forward (rrs-outer-F: 5'-CTCAGAACTAACGCTGGCGGCGCG-3'), reverse primers (rrs-outer-R: 5'-GGTTCGTTACTGAGGGTTAAAACCCCC-3') and 5 µL of the DNA from the animal urine sample. The assay was performed using the MJ Research PCT-200 Thermal Cycler (Bio-Rad, CA, USA) with the following cycling conditions: 95°C for 2 minutes; 40 cycles of 95°C for 10 seconds, 67°C for 15 seconds and 72°C for 30 seconds followed by a final extension at 72°C for 7 minutes. The second 25µl PCR reaction used 2 µL of the first PCR products in the same master mix as the first PCR, but containing 0.5 pmol each of inner forward (rrs-inner-F: 5'-CTGGCGGCGCGTCTTA-3') and inner reverse primers (rrs-inner-R: 5'-GTTTTCACACCTGACTTACA-3'). The cycling conditions of the second PCR were modified from those of the first PCR by reducing the annealing temperature from 67°C to 55°C. The expected 547-bp amplicons were visualised using 1.5% agarose gel electrophoresis. Then, the PCR products were purified from agarose gel using GenepHlowTM Gel/PCR Kit (Geneaid, New Taipei City, Taiwan). It was conducted as described in the manufacturer’s protocol. The purified products were sent to Bionics (South Korea) for Sanger sequencing.

The chromatogram results were inspected and edited to achieve consensus between the amplicon sequences from the forward and reverse sequencing primers using BioEdit Sequence Alignment Editor version 7.0.5.3. Maximum likelihood (ML) trees were reconstructed from the trimmed 443-nucleotide partial 16S rRNA gene alignments (from position 63 to 505 based on L. alexanderi GenBank accession number: NR_043047.1) on the General Time Reverse model to infer species and genetic relatedness using the Molecular Evolutionary Genetics Analysis (MEGA) software version 11 [2]. The number of base differences per sequence from averaging over all sequence pairs within each group was estimated. All sequences from this study (accession numbers: OQ446624-OQ446662) (Supplementary Table 1) and the 57 reference sequences of 40 *Leptospira* spp. acquired from GenBank data used for the analysis (Supplementary Table 2). The initial trees for the heuristic search were obtained automatically by applying Neighbor-Joining and BioNJ algorithms to a matrix of pairwise distances estimated using the Maximum Composite Likelihood (MCL) approach and selecting the topology with superior log likelihood value. A discrete Gamma distribution was used to model evolutionary rate differences among sites (5 categories, with the +G parameter set to 0.1284). The rate variation model allowed some sites to be evolutionarily invariable ([+I], 42.89% sites). The tree was drawn to scale, with branch lengths measured in substitutions per site. All positions with less than 95% site coverage were eliminated, and ambiguous bases were allowed at any position (partial deletion option). The phylogenetic tree was displayed and annotated using the Interactive Tree Of Life version 6.0 [3].

**Five pathogenic *Leptospira* clusters identification**

The 39/56 *Leptospira* PCR-screening-positive dogs and cats, which had accurate partial 16S rRNA sequences (each chromatogram peak confirmed by forward and reverse sequencing primer reads), were closely related to the P clade. The ML tree shows that the 39 sequences from infected cats and dogs were grouped into five clusters, called after the species name of the dominating reference sequences in each cluster except the last cluster using subclade name: Interrogans (n=18, 46%), Borgpetersenii (n=5, 13%), Weilii (n=2, 5%), Yasudae (n=6, 15%), and P2 (n=8, 21%) clusters. The unknown sequences in the first three clusters were assigned species corresponding to the cluster names, and they were assigned to Group 1 of the P1 (P1-1), which are common causes of human leptospirosis worldwide. Members in these clusters were grouped with a bootstrap higher than 50%. The sequences of the Yasudae cluster were grouped with less than 50% bootstrap support. They were assigned to Group 2 of the P1 (P1-2), which was isolated only from environmental samples. The last cluster contained sequences from several species in the P2. Among the undetermined species of P2, three groups can be observed: 1) *L. andrefontaineae*, *L. haakeii*, *L. hartskeerlii*, *L. selangorensis*, *L. venezuelensis*, and *L. wolffii*; 2) *L. sarikeiensis*, *L. semungkisensis*, and *L. langatensis*; and 3) *L. neocaledonica*, *L. saintgironsiae*, *L. koniamboensis*, *L. johnsonii*, and *L. licerasiae*. 6/8 (dogs and cats from Ranong and Prachuap Khiri Khan) were closely related to the *L. wolffii* group, and 2/8 (dog and cat from Tak and Ranong) were closely related to the *L. licerasiae* group. Within each cluster, a range of zero to eleven single-nucleotide polymorphisms (SNPs) were found as shown in Fig 1. Of 443 bp, none of the SNPs were found within the Borgpetersenii or Weilii clusters, while six, four and eleven SNPs were found in the Interrogans, Yasudae, and P2 subclade clusters, respectively.

**Fig 1**. Single Nucleotide Polymorphism found among pathogenic *Leptospira* spp. in Thai dogs and cats compared with reference sequences

**References**

1. Boonsilp S, Thaipadungpanit J, Amornchai P, Wuthiekanun V, Chierakul W, Limmathurotsakul D, et al. Molecular detection and speciation of pathogenic Leptospira spp. in blood from patients with culture-negative leptospirosis. BMC Infect Dis. 2011;11:338. Epub 20111213. doi: 10.1186/1471-2334-11-338. PubMed PMID: 22151687; PubMed Central PMCID: PMCPMC3297668.

2. Tamura K SG, Kumar S. MEGA 11: Molecular Evolutionary Genetics Analysis. Version 11 [software]. Molecular Biology and Evolution; 2021.

3. Letunic I, Bork P. Interactive Tree of Life (iTOL) v6: recent updates to the phylogenetic tree display and annotation tool. Nucleic Acids Res. 2024;52(W1):W78-w82. doi: 10.1093/nar/gkae268. PubMed PMID: 38613393; PubMed Central PMCID: PMCPMC11223838.
