# Supplementary material for: Pathogenic Leptospira species identified in dogs and cats during neutering in Thailand
Source: PLoS Negl Trop Dis. 2026 Feb 4;20(2):e0013421. doi: 10.1371/journal.pntd.0013421 (PMC12871963; doi:10.1371/journal.pntd.0013421)
Supplement: S2 File — (DOCX) [file pntd.0013421.s002.docx]

**S2 File** **Annual case and morbidity rate of study sites reported to the Department of Disease Control, Ministry of Public Health of Thailand, between 2019 and 2022**

Human leptospirosis is a communicable disease that is monitored under the National Disease Surveillance System (R506) of the Department of Disease Control, Ministry of Public Health, Thailand [1]. Leptospirosis cases were reported weekly via the R506 program without classification as suspected, probable or confirmed [2]. Patients were categorised by calendar year based on the date of onset, and the overall incidence rate per 100,000 population was calculated using annual population data [3]. The annual incidence of human leptospirosis reported in seven provinces of our study sites from 2019 to 2022 is described in the reported case, annual incidence, including the median, minimum, maximum, as well as the interquartile range (IQR, Q3-Q1) of morbidity for 4 years for each province, as shown in the table below.

|  |  | **Number of human leptospirosis cases**  **(incidence per 100,000 people)** | | | | **The average incidence over 4 years** | | |
| --- | --- | --- | --- | --- | --- | --- | --- | --- |
| **Region** | **Province** | **2019** | **2020** | **2021** | **2022** | **Median** | **Min-Max** | **IQR** |
| **Central** | Nakhon Sawan | 5 | 4 | 2 | 9 |  |  |  |
|  |  | (0.47) | (0.38) | (0.19) | (0.87) | 0.425 | 0.19-0.87 | 0.33-0.57 |
|  | Nakhon Pathom | 0 | 2 | 0 | 0 |  |  |  |
|  |  |  | (0.22) |  |  | 0 | 0-0.22 | 0-0.06 |
|  | Samut Sakhon | 0 | 0 | 0 | 0 |  |  |  |
| **Western** | Tak | 11 | 8 | 17 | 11 |  |  |  |
|  |  | (1.67) | (1.2) | (2.52) | (1.63) | 1.65 | 1.2-2.52 | 1.52-1.88 |
|  | Kanchanaburi | 1 | 5 | 0 | 7 |  |  |  |
|  |  | (0.11) | (0.56) |  | (0.78) | 0.335 | 0-0.78 | 0.08-0.62 |
|  | Prachuap Khiri Khan | 2 | 0 | 1 | 1 |  |  |  |
|  |  | (0.36) |  | (0.18) | (0.18) | 0.18 | 0-0.36 | 0.14-0.23 |
| **Southern** | Ranong | 46 | 57 | 83 | 135 |  |  |  |
|  |  | (23.88) | (29.4) | (42.68) | (69.38) | 36.04 | 23.88-69.38 | 28.02-49.36 |

**References**

1. Epidemiological surveillance disease data via the Program 506 of the Epidemiology Division, Department of Disease Control [accessed on July 24, 2025] retrieved from http://doe1.moph.go.th/surdata/index.php.

2. Department of Disease Control [Internet]. 2023. Dangerous communicable diseases, communicable diseases requiring surveillance, and syndromic surveillance [cited July 24, 2025]. Available from: https://ddc.moph.go.th/uploads/files/3181820230207100710.pdf [in Thai].

3. Sawangpol C, Aimyong N, Phosri A. Epidemiological changes in the incidence of human leptospirosis in Thailand: Findings from the National Disease Surveillance System from 2013 to 2022. Infect Dis Now. 2025 Jun 21;55(6):105108. doi: 10.1016/j.idnow.2025.105108. Epub ahead of print. PMID: 40550396.
