# Supplementary material for: Pathogenic Leptospira species identified in dogs and cats during neutering in Thailand
Source: PLoS Negl Trop Dis. 2026 Feb 4;20(2):e0013421. doi: 10.1371/journal.pntd.0013421 (PMC12871963; doi:10.1371/journal.pntd.0013421)
Supplement: S3 Table — (DOCX) [file pntd.0013421.s005.docx]

S3 Table Frequency of *Leptospira* groups in each animal by study site, age group, sex, and owned status

|  | **Dog** | | | | | | **Cat** | | | | | |  |
| --- | --- | --- | --- | --- | --- | --- | --- | --- | --- | --- | --- | --- | --- |
|  | **P1-1** | ***L. yasudae^b^*** | **P2** | **Unassigned species** | **Not infection** | **Total** | **P1-1** | ***L. yasudae^b^*** | **P2** | **Unassigned species** | **Not infection** | **Total** | |
|  | **(int-borg-wei)^a^**  **(%)** | **(%)** | **(%)** | **(%)** | **(%)** | **(%)** | **(int-borg-wei)^a^**  **(%)** | **(%)** | **(%)** | **(%)** | **(%)** | **(%)** | |
| **Animals**  **(n=567)** | 15 | 3 | 3 | 13 | 269 | 303 | 10 | 3 | 5 | 4 | 242 | 264 | |
|  | (11-2-2)(5.0) | (1.0) | (1.0) | (4.2) | (88.8) | (53.4) | (7-3-0)(3.8) | (1.1) | (1.9) | (1.5) | (91.7) | **(46.6)** | |
| **Study sites** |  |  |  |  |  |  |  |  |  |  |  |  | |
| Nakhon Pathom | 1 | 0 | 0 | 0 | 73 | 74 | 0 | 0 | 0 | 0 | 2 | 2 | |
| (n=76) | (0-1-0)(1.4) |  |  |  | (98.6) | (97.4) |  |  |  |  | (100) | (2.6) | |
| Kanchanaburi | 1 | 0 | 0 | 0 | 21 | 22 | 1 | 0 | 0 | 0 | 20 | 21 | |
| (n=43) | (0-0-1)(4.6) |  |  |  | (95.4) | (51.2) | (1-0-0)(4.8) |  |  |  | (95.2) | (48.9) | |
| Prachuap Khiri Khan | 2 | 3 | 1 | 12 | 37 | 55 | 1 | 2 | 3 | 3 | 38 | 47 | |
| (n=102) | (2-0-0)(3.6) | (5.5) | (1.8) | (21.8) | (67.3) | (53.9) | (1-0-0)(2.1) | (4.2) | (6.4) | (6.4) | (80.9) | (46.1) | |
| Ranong | 7 | 0 | 1 | 1 | 99 | 108 | 4 | 1 | 2 | 1 | 122 | 130 | |
| (n=238) | (5-1-1)(6.5) |  | (0.9) | (0.9) | (91.7) | (45.4) | (1-3-0)(3.1) | (0.8) | (1.5) | (0.8) | (93.8) | (54.6) | |
| Tak | 4 | 0 | 1 | 0 | 19 | 24 | 4 | 0 | 0 | 0 | 28 | 32 | |
| (n=56) | (4-0-0)(16.7) |  | (4.1) |  | (79.2) | (42.9) | (4-0-0)(12.5) |  |  |  | (87.5) | (57.1) | |
| Nakhon Sawan | 0 | 0 | 0 | 0 | 20 | 20 | 0 | 0 | 0 | 0 | 20 | 20 | |
| (n=40) |  |  |  |  | (100) | (50.0) |  |  |  |  | (100) | (50.0) | |
| Samut Sakhon | 0 | 0 | 0 | 0 | 0 | 0 | 0 | 0 | 0 | 0 | 12 | 12 | |
| (n=12) |  |  |  |  |  |  |  |  |  |  | (100) | (100) | |
| **Sex** |  |  |  |  |  |  |  |  |  |  |  |  | |
| Male | 3 | 1 | 0 | 6 | 104 | 114 | 1 | 1 | 2 | 1 | 62 | 67 | |
| (n=181) | (1-1-1)(2.6) | (0.9) |  | (5.3) | (91.2) | (63.0) | (0-1-0)(1.5) | (1.5) | (3.0) | (1.5) | (92.5) | (37.0) | |
| Female | 12 | 2 | 3 | 6 | 156 | 179 | 9 | 2 | 3 | 3 | 157 | 174 | |
| (n=353) | (10-1-1)(6.7) | (1.1) | (1.7) | (3.4) | (87.1) | (50.7) | (7-2-0)(5.2) | (1.2) | (1.7) | (1.7) | (90.2) | (49.3) | |
| Missing | 0 | 0 | 0 | 1 | 9 | 10 | 0 | 0 | 0 | 0 | 0 | 0 | |
| (n=33) |  |  |  | (10.00) | (90.00) | (30.3) |  |  |  |  |  |  | |
| **Age group** |  |  |  |  |  |  |  |  |  |  |  |  | |
| Junior | 0 | 1 | 1 | 0 | 31 | 33 | 0 | 0 | 0 | 0 | 13 | 13 | |
| (n=46) |  | (3.0) | (3.0) |  | (94.0) | (71.7) |  |  |  |  | (100) | (28.3) | |
| Adult | 15 | 2 | 2 | 13 | 238 | 270 | 10 | 3 | 5 | 4 | 229 | 251 | |
| (n=521) | (11-2-2)(5.6) | (0.7) | (0.7) | (4.8) | (88.2) | (51.8) | (7-3-0)(4.0) | (1.2) | (2.0) | (1.6) | (91.2) | (48.2) | |
| **Status** |  |  |  |  |  |  |  |  |  |  |  |  | |
| Owned | 5 | 1 | 1 | 4 | 97 | 108 | 4 | 1 | 4 | 3 | 151 | 163 | |
| (n=271) | (3-2-0)(4.6) | (0.9) | (0.9) | (3.7) | (89.9) | (39.9) | (2-2-0)(2.5) | (0.6) | (2.5) | (1.8) | (92.6) | (60.1) | |
| Free-roaming | 10 | 2 | 2 | 9 | 172 | 195 | 6 | 2 | 1 | 1 | 91 | 101 | |
| (n=296) | (8-0-2)(5.2) | (1.0) | (1.0) | (4.6) | (88.2) | (65.9) | (5-1-0)(5.9) | (2.0) | (1.0) | (1.0) | (90.1) | (34.1) | |

^a^Proportion of three species in abbreviation of *L. interorgan*s, *L. borgpetersenii* and *L. weilii*, *^b^L. yasudae* is grouped into the P1-2
