# Supplementary material for: Pathogenic Leptospira species identified in dogs and cats during neutering in Thailand
Source: PLoS Negl Trop Dis. 2026 Feb 4;20(2):e0013421. doi: 10.1371/journal.pntd.0013421 (PMC12871963; doi:10.1371/journal.pntd.0013421)
Supplement: Attachment — Submitted filename: ResponseToReviewers_20260105_V5.3.docx [file pntd.0013421.s008.docx]

**Manuscript PNTD-D-25-01286**

**Response to reviewers**

Dear Dr Beechler and Professor Blacksell

Thank you for giving us the opportunity to submit a revised draft of the manuscript “Pathogenic *Leptospira* species identified in dogs and cats during neutering in Thailand” for publication in the *PLOS Neglected Tropical Diseases*. We appreciate the time and effort you and the reviewers dedicated to providing feedback on our manuscript, and we are grateful for the insightful comments and valuable improvements to our paper. We have incorporated most of the reviewers' suggestions. Those changes are underlined within the manuscript. Please see below, in blue, for a point-by-point response to the reviewers’ comments and concerns. All page numbers refer to the revised manuscript file with tracked changes.

**Reviewers’ Comments to the Authors:**

**Reviewer 1**

**Additional Editor Comments (if provided):**

 Two reviewers have several minor suggested revisions on things that should be clarified in the methods or discussed briefly and/or some details that may be better placed in supplement. Please respond to their concerns and queries.

Author response: Thank you for summarising the reviewers' feedback. We have carefully addressed all concerns and queries raised by the reviewers. As suggested, we have clarified specific points in the Methods section and moved detailed methodological descriptions and secondary results to the Supplementary Material to improve the flow of the main manuscript. A point-by-point response to the reviewers is below.

**Journal Requirements:**

If the reviewer comments include a recommendation to cite specific previously published works, please review and evaluate these publications to determine whether they are relevant and should be cited. There is no requirement to cite these works unless the editor has indicated otherwise.

Author response: Thank you for clarifying. We acknowledge it.

**Reviewers' comments:**

 Reviewer's Responses to Questions

**Key Review Criteria Required for Acceptance?**
As you describe the new analyses required for acceptance, please consider the following:
**Methods**
-Are the objectives of the study clearly articulated with a clear testable hypothesis stated?
-Is the study design appropriate to address the stated objectives?
-Is the population clearly described and appropriate for the hypothesis being tested?
-Is the sample size sufficient to ensure adequate power to address the hypothesis being tested?
-Were correct statistical analysis used to support conclusions?
-Are there concerns about ethical or regulatory requirements being met?

**Reviewer #1**: The article has the aim to investigate *Leptospira* infection in dogs and cats in three regions in Thailand and to provide epidemiological data for the purpose of control and prevention of the infection.
The abstract gives the methods used and results of the study. The title recalls the key features of the article and does not add anything more to the findings of the study.
The references are cited correctly and are mostly recent (20 out of 98 are dated before 2015).
The introduction briefly summarizes the current knowledge about the epidemiology of leptospirosis, citing papers reporting data from different countries and referred on studies on dogs and cats.
The introductive part clearly defines the purpose of the research as indicated in the abstract.
The paper presents a cross-sectional study based on samples collected in three different regions in Thailand.
The study design, the criteria for sampling and the methods used are clearly described. The molecular tests are known and supported by references.
The statistical methods are clearly defined.

Author response:

We sincerely thank you, Reviewer#1, for your positive assessments of the following sections: Abstract, Introduction, and Methods (and the study design). No changes were necessary.

**Reviewer #2**: The objectives, experimental design, sampling, and statistical model are compatible with the proposed study.

Author response:

We sincerely thank you, Reviewer#2, for your positive assessment of the methodology. No changes were necessary.

However, a few details regarding the sampling require further information (specifically on the intermittency of bacterial shedding and sample preservation).

Reviewer#2 highlighted this important issue, which we were previously unaware of, and addressed it. To clarify these two points, we have added sentences in the “Methods” section as follows:

Line 155-159	“… We collected a single urine sample from each animal. Available urine volume in the urine blader was collected into a new sterile 50 ml tube and kept on ice upon collection. The urine samples were transported to laboratories at cool temperatures (2°C to 6°C) and then stored at -80°C until used for DNA preparation. …”

Reviewer #3: Yes
Yes
N/A
Yes
Yes
Yes

Author response:

There were no specific comments from Reviewer #3 in this section. However, all comments of Reviewer #3 will be addressed later in the last section.

**Results**
-Does the analysis presented match the analysis plan?
-Are the results clearly and completely presented?
-Are the figures (Tables, Images) of sufficient quality for clarity?
**Reviewer #1**: The data are presented appropriately: tables and figures are clear with titles, columns and rows labelled correctly and clearly.
The text summarizes briefly the contents of the tables but it is not repetitive.

Author response:

We sincerely thank you, Reviewer #1, for your positive assessments of the tables and figures. No changes were necessary.

**Reviewer #2**: The results are compatible with the proposed hypothesis; however, due to the characteristics of the clinical stage of the disease (the leptospiruria phase), intermittent bacterial shedding in the urine may occur. Therefore, the authors need to, at a minimum, mention that the results may be underestimated.

Author response:

We sincerely thank you, Reviewer#2, for highlighting the underestimated results due to missing leptospiremia cases, an important issue we were previously unaware of, and for addressing it. To clarify these two points, we have added sentences in the “Results” section as follows:

Line 206-209	“… The infection rates are likely underestimated because the study design only detects chronic carriers (leptospiruria) and is subject to the inherent challenge of intermittent bacterial shedding in the urine.”

**Reviewer #3**: Please ref the reviewer comments attached

Author response:

All comments of Reviewer #3 will be addressed later in the last section.

**Conclusions**
-Are the conclusions supported by the data presented?
-Are the limitations of analysis clearly described?
-Do the authors discuss how these data can be helpful to advance our understanding of the topic under study?
-Is public health relevance addressed?

**Reviewer #1**: In the first part of the discussion, a number of papers on the prevalence of Leptospira infection in different countries are cited. The authors are conscious (see lines 327-328) that comparisons between results of different studies are not always possible and that variations depends from a number of factors. Moreover, the authors must be careful with the use of term “prevalence” when they refer to the figures from countries they have considered.

Author response:

We sincerely thank you, Reviewer #1, for highlighting the use of “prevalence”. We have checked all the references again, ensuring the comparison of *Leptospira* carriage prevalence (in kidney or urine samples) among dogs and cats between our data and previous reports in Thailand and other countries. Please note that the paragraph was revised in response to comments from Reviewer #3 as well. The revised text is as follows:

Line 274-285	“The urinary shedding prevalence in this study was slightly higher than that reported in previous Thai studies [30-32, 43]. In dogs, our findings were comparable to those from Malaysia, the USA and several European nations [34, 38, 41, 45, 47], but remain lower than the high-burden regions of South Asia, South America and Iran, where the prevalences ranged between 10.6% and 97.4% [33, 37, 39, 42, 46, 48, 50, 53, 56]. In cats, our prevalence fits within the broad international range of 0.3% to 12.9% reported in several countries of Asia, Europe, Latin America and the Caribbean [49, 58, 59, 61-67, 69], contrasting with the strikingly high prevalence reported in Taiwan (67.8%)[60]. Variations in the urinary shedding prevalence may be attributed to geographical location, study population, sample size, season, and screening techniques. The application of molecular methods to specifically identify pathogenic *Leptospira* DNA in clinically healthy carriers provides a crucial tool for monitoring transmission risks from these animals and informing targeted control strategies.”

**Reviewer #2**: The conclusions are consistent with the stated objectives.

Author response:

We sincerely thank you, Reviewer#2, for your positive assessment of the conclusion. No changes were necessary.

**Reviewer #3**: Please refer to the reviewer comments attached.

Author response:

All comments of Reviewer #3 will be addressed later in the last section.

**Editorial and Data Presentation Modifications?**
Use this section for editorial suggestions as well as relatively minor modifications of existing data that would enhance clarity. If the only modifications needed are minor and/or editorial, you may wish to recommend “Minor Revision” or “Accept”.

Reviewer #1: (No Response)

Reviewer #2: No

Reviewer #3: Please refer to the reviewer comments attached.

**Summary and General Comments**
Use this section to provide overall comments, discuss strengths/weaknesses of the study, novelty, significance, general execution and scholarship. You may also include additional comments for the author, including concerns about dual publication, research ethics, or publication ethics. If requesting major revision, please articulate the new experiments that are needed.

**Reviewer #1**: The study, despite the limits specified, gives valuable insights suggesting region-specific mitigation strategies to reduce the risk of Leptospira transmission.

Author response:

We sincerely thank you, Reviewer#1, for your positive assessment.

**Reviewer #2**: To the Authors,

First and foremost, I would like to congratulate you on the results obtained, particularly given the substantial sample size. The results are strongly supported by the techniques employed and are, likewise, carefully discussed.

Author response:

We sincerely thank you, Reviewer#2, for your positive assessment.

However, a few minor observations and questions need to be clarified in the manuscript:
Positive PCR results from urine samples indicate that the animal is in the leptospiruria phase. However, as bacterial shedding is intermittent during this phase—that is, inconsistent over time—negative results do not allow one to conclude that the patient is not a carrier. This information should be included in the discussion, noting that the results may be underestimated; in other words, the detection frequency could be higher than reported.

Author response:

We sincerely thank you, Reviewer #2, for your concern about the intermittent shedding of bacteria during the leptospiruria phase, which could lead to underestimation of the study's results. We agree that negative PCR results from a single sample collection cannot rule out *Leptospira* carriage in the animals. Therefore, we have discussed further on this issue in the “Discussion” section as follows (two involving references were also added):

Line 353-359	“… Second, the reported prevalence likely underestimates the true infection rate. As our study focused on urinary shedding (leptospiruria) to identify chronic carriers, we did not perform blood sample investigation; consequently, animals in the acute phase of infection (where the pathogen is present in the blood but not yet shed in the urine) were not captured. Furthermore, because *Leptospira* are shed intermittently [25, 42], a single-point urine collection may have yielded false-negative results for some carriers. ...”

References:

25. Sant'Anna da Costa R, N Di Azevedo MI, Dos Santos Baptista Borges AL, Carvalho-Costa FA, Martins G, Lilenbaum W. Persistent High Leptospiral Shedding by Asymptomatic Dogs in Endemic Areas Triggers a Serious Public Health Concern. Animals (Basel). 2021;11(4):937. doi: 10.3390/ani11040937. PMID: 33810226; PMCID: PMC8065945.

42. Miotto BA, Guilloux AGA, Tozzi BF, Moreno LZ, da Hora AS, Dias RA, Heinemann MB, Moreno AM, Filho AFS, Lilenbaum W, Hagiwara MK. Prospective study of canine leptospirosis in shelter and stray dog populations: Identification of chronic carriers and different *Leptospira* species infecting dogs. PLoS One. 2018 ;13(7):e0200384. doi: 10.1371/journal.pone.0200384. PMID: 29995963; PMCID: PMC6040711.

Were any of the animals under preoperative treatment (antibiotic therapy)? The administration of antibiotics prior to sample collection may lead to false-negative PCR results.

Author response:

We sincerely thank you, Reviewer #2, for your concern about other factors that might lead to false PCR negatives, such as preoperative treatment. We have clarified this issue further in the Discussion section as follows:

Line 359-361	“… Other factors also contribute to this underestimation, including potential false PCR negatives due to antibiotic administration before urine collection (a factor we could not fully control, though our neutering protocol did not use antibiotics). ...”

Was any buffer (e.g., PBS) used to stabilize the urinary pH immediately after collection? DNA extraction from urine can be interfered with by several factors and may even be rendered unviable. As urine pH is acidic, it is important to mix it with PBS immediately after collection to neutralize it.

Author response:

We sincerely thank you, Reviewer #2, for your concern that DNA extraction might be affected if urinary pH is not stabilised during urine collection. Due to labour, time, and space constraints, we could not perform a complex process at the field site. We only preserved cells by maintaining a cold chain at 2-4 °C. We have added this limitation in the Discussion section as follows:

Line 361-365	“…Third, logistical constraints impacted molecular identification. Due to limited field resources, samples were maintained at 2°C–6°C without pH neutralisation. The resulting exposure to potentially acidic urine, particularly in samples requiring prolonged transport (>24 hours) from distant sites, may have caused DNA degradation and reduced diagnostic sensitivity. …”

What was the time elapsed between sample collection and DNA extraction? If DNA is not extracted on the same day, it is recommended that the urine (with stabilised pH) be stored at 5°C and the DNA extracted within the next 24 hours. What procedure was adopted in the study?

Author response:

We sincerely thank you, Reviewer #2, for your concern time elapsed between sample collection and DNA extraction. We have discussed the limitations in the Discussion section in response to the comment above.

**Reviewer #3**

**General:**

This study provides valuable One Health data on asymptomatic *Leptospira* infections in dogs and cats, with the novel detection of *L. yasudae* and P2 clade species adding to its significance. The methodology is sound, and the results are clearly supported by the data. The main areas for improvement are reducing overly descriptive sections in the Introduction and Discussion and moving some technical details from the Methods/Results to the Supplementary Material for clarity. Overall, the manuscript is strong and requires only minor revisions before acceptance.

Author response:

We sincerely thank you, Reviewer #3, for your valuable and insightful comments and suggestions. We have responded to each comment below.

**Abstract**

**Line 26:** Revise phrasing → *“Pathogenic species of the genus Leptospira cause an underdiagnosed zoonosis…”* for scientific accuracy.

Revised as Line 26-27	“Pathogenic species of the genus *Leptospira* cause an underdiagnosed zoonosis in humans and animals called leptospirosis. …”

**Line 27–28:** Reword → *“Animal reservoirs often remain asymptomatic yet shed the bacteria in urine…”* to improve clarity.

Revised as Line 27-28	“Animal reservoirs often remain asymptomatic yet shed the active spirochete in urine…”

**Line 30:** Replace *“leading to unawareness of infections”* with *“resulting in unrecognized infections”*.

Revised. Line 29-30	“… Asymptomatic leptospirosis in human companions, such as dogs and cats, resulting in unrecognised infections…”

**Line 32–33:** Consider stating the knowledge gap in Thailand to highlight novelty.

To highlight the novelty, we added the following sentence:

Line 30-33	“…Crucially, the current lack of molecular epidemiology data on *Leptospira* among companion animals in Thailand underscores the urgent need to investigate transmission dynamics for effective regional control.”

**Line 34:** Simplify → *“We investigated the prevalence of Leptospira infection in cats and dogs in Thailand.”*

We try to balance the key information and simplify it per your kind suggestion. We revised the sentence as follows:

Line 34-35	“We investigated the prevalence of *Leptospira* infection in cats and dogs during neutering in seven provinces across Thailand. …”

**Line 36–37:** Replace *“screened for Leptospira’s DNA”* with *“screened for Leptospira DNA by PCR targeting the rrs gene.”*

Revised as Line 35-36	“… The urine samples were screened for *Leptospira* DNA by PCR targeting the *rrs* gene…”

**Line 37–41:** Present prevalence more clearly: first overall (56/567, 9.9%), then breakdown by dogs (11.2%) and cats (8.3%).

Revised as Line 37-38	“… The 56/567 (9.9%) animals were positive for *Leptospira* in the Pathogen clade, including 34/303 (11.2%) dogs and 22/264 (8.3%) cats. …”

**Line 39–41:** Emphasize novelty → *“This represents the first detection of L. yasudae in companion animals in Thailand.”*

We revised those sentences corresponding to your kind suggestion as follows:

Line 38-41	“… The partial *rrs* gene analysis identified *L. interrogans*, *L. weilii*, and *L. borgpetersenii* (4.4%) as well as Pathogen subclade 2 species (1.4%). Notably, this study reports the first molecular detection of *L. yasudae* (1.0%) in companion animals in Thailand.”

**Line 42–45:** Replace casual phrase *“probably living under the same roof as us”* with a more formal version: *“These companion animals, often living in close contact with humans, may contribute to daily transmission risks.”*

Revised as Line 44-45	“… These companion animals, often living in close contact with human, may contribute to daily risks. …”.

**Line 46–47:** Strengthen conclusion → *“Enhanced surveillance and vaccination of dogs and cats, combined with public awareness campaigns, may reduce human infection risks.”*

We revised according to your suggestion as follows:

Line 45-47	“…Therefore, enhanced surveillance and vaccination programs for dogs and cats, coupled with targeted public awareness campaigns, are critical for mitigating the risk of human infections.”

**Line 48:** Keywords are fine; consider adding *“molecular epidemiology”* for better indexing.

Added as Line 48-49	“**Keywords:** *Leptospira*, urine, asymptomatic, dog, cat, Thailand, leptospirosis, molecular epidemiology”.

**Introduction:**

**Overall:**

- Streamline taxonomy and prevalence details.
- Ensure statistics are current and contextualized.
- Reorganize flow: global context → Thailand data → pets as reservoirs → knowledge gap → study aim.

Thank you for your insightful comments. We will revise as the details below.

**Line 68–74:**

- Revise wording for clarity (*“The genus Leptospira comprises Gram-negative spirochetes…”*).

Revised as Line 68	“The genus *Leptospira* comprises Gram-negative spirochetes.” .

- Merge sentences on carriers and transmission to reduce repetition.

We have revised the text to reduce the repetition as follows:

Line 70-73	“… Many mammals, including rodents, dogs, cats, pigs, bats, and cattle, serve as reservoir hosts for spirochetes, harbouring the bacteria in their proximal renal tubules without showing clinical signs [3, 4]. They excrete living bacteria in their urine, which can transmit the disease through direct or indirect exposure to contaminated environments [3-5]. …”

**Line 75–85:**

- Taxonomy section is too detailed; condense. Keep only clinically relevant species (e.g., *L. interrogans, L. borgpetersenii, L. weilii, L. yasudae*).

We focus more on pathogen clades and mention only three pathogen species names, which are clinically relevant, as follows:

Line 75-78	“Of 68 species in the genus *Leptospira*, 40 are categorised into the Pathogen (P) clade, based on whole genome sequence analysis [6-12]. The P clade comprises two subclades (P1 and P2). Of the 19 species in subclade P1, eight (including *L. interrogans*, *L. weilii* and *L. borgpetersenii*) are key species that cause leptospirosis worldwide. …”

- Avoid long lists of rarely reported species in the Introduction.

We keep only three species names as examples for each pathogen group to avoid long lists, as follows:

Line 78-82	“… The other eleven species (including *L. alstonii, L. kmetyi* and *L. yasudae*) are recently discovered species isolated from environmental samples, with no evidence of human or mammalian infections. Of 21 species of the P2, five (including *L. wolffii* and *L. licerasiae*) have been known as the intermediate group, causing mild leptospirosis in mammals and humans [11, 13-17].”

**Line 86–95:**

- Update global burden statistics (current WHO/GBD).

We only find that these numbers are the most up-to-date, having been estimated using data from 1970 to 2008 and reported in 2015 by Costa *et. al.* (Global Morbidity and Mortality of Leptospirosis: A systematic Review). We revised to be clear as follows:

Line 84-85	“… In 2015, Costa et al. estimated over 1 million global human infections and more than 58,000 fatal cases (using data from 1970 to 2008) [18]. …”

- Simplify risk factors sentences; split into two shorter sentences for readability.

Revised as per your suggestion as follows:

Line 90-94	“… Computational models showed that leptospirosis transmission is amplified by environmental changes, specifically increased rainfall and flood exposure. Furthermore, using diverse and potentially contaminated household water sources and occupational contact with moist soil, environmental water, or infected animals significantly raise the risk for all hosts (animals and humans) [21-23].”

**Line 96–105:**

- Strengthen rationale for companion animals as reservoirs.

Revised as your suggestion as follows:

Line 99-102	“… Companion animals, especially free-roaming dogs and cats, act as a critical bridge for *Leptospira* transmission. They acquire pathogens from primary reservoirs, such as rodents and contaminated environments, and then introduce them into households, where the disease can be passed to owners through close, intimate exposures. …”

- Reword casual phrasing (*“bring pathogens from outside into houses” → “may introduce pathogens into households”*).

Rephrased as follows:

Line 100-101	“They acquire pathogens from primary reservoirs, such as rodents and contaminated environments, and then introduce them into households,…”

- Condense pet population statistics (Line 102–103). Suggest including “Athapattu T, Fernando R, Abayawansha R, Fernando P, Fuward M, Samarakoon N, et al. Carrier Status of *Leptospira* spp. in Healthy Companion Dogs in Sri Lanka. Vector Borne Zoonotic Dis. 2022;22(2):93-100. doi: 10.1089/vbz.2021.0065.”

Thank you for this suggestion. We have condensed the section on pet population statistics as requested. Regarding the suggested reference (Athapattu et al., 2022), we carefully reviewed it but decided not to include it in this specific sentence (Lines 102–103). Our text focuses exclusively on pet demographics and free-roaming animal statistics within Thailand, whereas the suggested study reports data from Sri Lanka. To maintain geographical accuracy and avoid confusing the reader, we restricted the citations in this paragraph to Thai national reports and local studies.

Line 102-105	“… In Thailand, the pet population was estimated at over 12 million between 2019 and 2020, with a high-risk group of approximately one million free-roaming animals [26-29]. The prevalence of *Leptospira* carriage in free-roaming and client-owned animals varies across the country’s geographic regions [30-32].”

**Line 106–113:**

- Global prevalence numbers (97% dogs, 68% cats) seem like an inflated clarify method (PCR vs serology).

We had presented the maximum infections. We have reported the median rate and IQR, including clarifying the investigation methods in this revision, as follows:

Line 106-108	“*Leptospira* infections (symptomatic or asymptomatic) have been reported in many countries using PCR or culture, with median rates of 7.3% (IQR=4.4-15.7%) in dogs and 6.3% (IQR=3.3-22.1%) in cats since 2003 [30-70]. …”

- Line 107 citation of references seems to be incorrect, isn’t it 30, 70? recheck.

We have checked. It is correct. In this sentence, we cited all 41 references from numbers 30 to 70.

**Line 114–117:**

- Knowledge gap statement (“scarce in Thailand”) should be moved earlier for emphasis.

We have redrafted to emphasise the gap at the beginning of the last paragraph of the introduction, per your suggestion, as shown below.

Line 106-110	“… *Leptospira* infections (symptomatic or asymptomatic) have been reported in many countries using PCR or culture, with median rates of 7.3% (IQR=4.4-15.7%) in dogs and 6.3% (IQR=3.3-22.1%) in cats since 2003 [30-70]. However, cross-sectional studies of asymptomatic *Leptospira* infections and carriages in dogs and cats in Thailand are scarce [31, 32]. …”

- Objective sentences should be tightened for clarity and impact.

We have redrafted the study objective as follows:

Line 116-119	“… In this study, we aimed to determine the prevalence of *Leptospira* carriage in dogs and cats across three regions of Thailand, establishing current molecular epidemiological data essential for directly informing targeted leptospirosis control strategies.”

**Methods:**

Methods are rigorous and reproducible but too detailed in technical parameters. Streamlining main text while moving exhaustive PCR/phylogenetic details to Supplementary would improve readability and fit *PLOS NTDs* style.

**Ethics Statement**

**Line 120–127**

- Well written and includes both IACUC and Biosafety approvals.

Thank you for your kind compliment.

- Clarify whether animal welfare guidelines (e.g., ARRIVE, OIE, or national standards) were followed. PLOS journals often require explicit mention of compliance with international standards.

We have added “The national standard's animal welfare guidelines were followed” (Line 124-125) in the Ethics statement section.

- Consider condensing slightly: consent procedures could be summarized in fewer sentences.

We have condensed the consent procedures into 2 sentences as follows:

Line 125-128	“… Written informed consent for participation was obtained from the animals' owners. For unowned or free-roaming dogs and cats collected from public areas (such as temples and schools), informed consent for a sample collection was obtained from the heads of each community. …”

**Sample Size Calculation**

**Line 128–133**

- Appropriate approach using ProMESA software.

Thank you.

- Relative error of 2% seems unusually strict and may inflate sample size requirements. Clarify why this threshold was chosen.

We are sorry for the confusion. Since we assume an infection prevalence of 0.1 (10%) with a relative error of 0.2, the results will have a precision of ±0.02 (±2%) for dogs, for example. ProMESA generally recommends an acceptable relative error of ≤ 0.2. We use the maximum precision range given the unknown prevalence of infections in the study regions. To clarify this, we have revised the paragraph as follows:

Line 132-137	“Sample size estimation was performed using ProMESA software version 2.3 (EpiCentre, Massey University, New Zealand) based on the previously reported leptospirosis prevalence at 10% [31] of dogs and 0.8% of cats [32] at a 95% confidence interval and a 0.2 acceptable relative error. We used the maximum of the generally recommended relative error as the unknown prevalence in the study regions. This resulted in a minimum sample size of 223 dogs and 236 cats, which was required for the study.”

**Study Population**

**Line 134–141**

- Wording “dogs and cats that look healthy” → should be *“clinically healthy dogs and cats”*.

Changed as Line 139.

- Ownership categories should be standardized (e.g., “owned” vs. “free-roaming/unowned”).

Changed as Line 141.

- Suggestion: Add numbers by group (owned vs free-roaming) if available. This will be useful for interpretation later.

We have added the number, as follows:

Line 140-141	“… All owned (n=271) and free-roaming/unowned (n=296) dogs and cats were included in this study. …”

**Line 138–140:**

- Province listing is correct but long; consider replacing with *“…across seven provinces in central, western, and southern Thailand”* and keep full details in Fig. 1 or Supplementary Table.

Changed as follows:

Line 142-143	“… The neutering program was carried out across seven provinces in central, western, and southern Thailand (Fig 1). The sex and estimated age of each animal were recorded.”

Line 145-151 “Fig 1. Geographic distribution of dog and cat Leptospira infections (Panel A) and median annual human leptospirosis incidence (Panel B) in seven provinces of Thailand: Nakhon Sawan, Nakhon Pathom, and Samut Sakhon (Central); Tak, Kanchanaburi, and Prachuap Khiri Khan (West); and Ranong (South). Infection frequencies in animals were identified using PCR and sequencing. Human incidence data (per 100,000 population) were reported by the Ministry of Public Health. Darker shades indicate higher prevalence/incidence. Base map shapefiles were obtained from The Humanitarian Data Exchange (sourced from the Royal Thai Survey Department)”

**Urine Collection and DNA Extraction**

**Line 154–165**

- Volume range (dogs: 1–370 mL) is extremely wide; clarify whether very large outliers are from larger animals or pooled samples.

We have clarified as follows:

Line 159-160	“… Collected urine specimens’ volume ranged from 1-370 ml (median = 42 ml) in dogs and 0.8–108 ml (median = 9.5 ml) in cats, which depended on each animal’s size. …”

- Specify how long samples were stored before processing. This has effect on DNA degradation.

We improved the Method section and added the limitation in the discussion as recommended by Reviewer#3 and Reviewer#2 as follows:

Line 156-159 (Method)	“… Available urine volume in the urine blader was collected into a new sterile 50 ml tube and kept on ice upon collection. The urine samples were transported to laboratories at cool temperatures (2°C to 6°C) and then stored at -80°C until used for DNA preparation. …”

Line 361-365 (Discussion)	“… Third, logistical constraints impacted molecular identification. Due to limited field resources, samples were maintained at 2°C–6°C without pH neutralisation. The resulting exposure to potentially acidic urine, particularly in samples requiring prolonged transport (>24 hours) from distant sites, may have caused DNA degradation and reduced diagnostic sensitivity. …”

**PCR and Sequencing**

**Line 166–187**

- Methods are detailed, but overly technical for an Introduction-to-Methods section. PCR primer sequences and thermocycler details are usually moved to Supplementary Methods.

We have moved the details of PCR to a new Supplementary Document (Supplementary Document 1)

- The primer names could be formatted in italics or in Supplementary Data for clarity.

We have moved the PCR details to the Supplementary Document 1. And we condense this section as follows:

Line 170-176	“The nested PCR assay targeting the partial 16S rRNA sequences of the P1 and P2 was conducted as described previously [72], with modifications as described in Supplementary Document 1. The expected 547-bp amplicons were visualised using 1.5% agarose gel electrophoresis. Then, the PCR products were purified from agarose gel using GenepHlow Gel/PCR Kit (Geneaid, New Taipei City, Taiwan). It was conducted as described in the manufacturer’s protocol. The purified products were sent to Bionics (South Korea) for the Sanger DNA Sequencing.”

**Line 187:** “would be sent to Bionics” → rephrase to *“were sent to Bionics (South Korea) for Sanger sequencing.”*

Changed as Line 175-176	“… The purified products were sent to Bionics (South Korea) for the Sanger DNA Sequencing.”

**Phylogenetic Analysis**

**Line 188–207**

- Description is too detailed for main Methods. Readers need to know:
  - Alignment length, Model used and Software version.

Thank you for your suggestions.

- Details on gamma distribution parameters (+G = 0.1284) and invariant sites (42.89%) can be shortened or placed in the Supplementary Material.

We have moved the details of phylogenetic tree reconstructions and analysis to Supplementary Document 1

- Condense to ~5 sentences in main text; move parameter-heavy details to Supplementary Table.

We have moved the details of phylogenetic tree reconstructions and analysis to Supplementary Document 1 and condensed the whole section into five sentences as follows:

Line 177-185	“The chromatogram results were inspected and edited for consensus using BioEdit Sequence Alignment Editor version 7.0.5.3. Maximum likelihood trees were reconstructed from the trimmed 443-nucleotide partial 16S rRNA gene alignments (from position 63 to 505 based on L. alexanderi GenBank accession number: NR_043047.1) using MEGA software version 11 [73]. The details of the algorithm used for the analysis were described in the Supplementary Document 1. All sequences from this study (accession numbers: OQ446624-OQ446662)(Supplementary Table 1) and the 57 reference sequences of 40 Leptospira spp. acquired from GenBank data used for the analysis, (Supplementary Table 2). The phylogenetic tree was displayed and annotated using the Interactive Tree Of Life version 6.0 [74].”

**Statistical Analysis**

**Line 208–215**

- Clear and appropriate (ORs with small sample adjustment, 95% CIs).

Thank you.

- Mention significance threshold (e.g., *p* < 0.05).

We have added as follows.

Line 191-194	“… Small-sample adjustments were applied to both OR estimates and CIs (using a normal approximation), and p-values were derived using Fisher's exact test. Statistical significance was defined as a p-value < 0.05 and a 95% CI that did not include 1. …”

- Clarify whether multiple testing corrections were applied.

We thank you for this important question. We want to clarify that we did not apply multiple-testing corrections (such as the Bonferroni correction) to this analysis. Our study was designed as an exploratory analysis using univariate methods to identify potential risk factors associated with *Leptospira* infection. In this epidemiological context, our primary goal was estimation rather than strict hypothesis testing. We assessed each factor (sex, age, ownership, study site) independently to understand its individual contribution to risk. We presented Odds Ratios (ORs) alongside 95% Confidence Intervals (CIs) to clearly communicate the magnitude of associations and the precision of our estimates. As noted by Rothman (1990) and others [1] in epidemiology, applying multiple-comparison adjustments in exploratory studies can be counterproductive, as it inflates Type II errors (false negatives) and may lead to the dismissal of potentially important biological associations. By reporting the unadjusted estimates and confidence intervals, we allow readers to assess the strength of the evidence for each factor on its own merits.

^1^Rothman, K. J. (1990). No adjustments are needed for multiple comparisons. Epidemiology, 1(1), 43-46.

Therefore, we have clarified this point by adding the following sentence.

Line 194-195	“… Adjustments for multiple comparisons were not applied, as the primary objective was to estimate the magnitude of associations rather than strictly test hypotheses. …”

- Good to note mapping was done using Excel but could also specify GIS software if used (Excel maps may raise questions about precision).

Regarding the mapping software, we acknowledge that GIS software offers superior precision for complex spatial analysis. In addition to the PLOS Neglected Tropical Diseases Staff concerning the copyright issue using the Excel chart, we have created Thai maps based on the shape files from the Royal Thai Survey Department using ‘ggplot’ in R as described in the Method section as follows:

Line 196-199	“… Finally, the geographical distribution of *Leptospira* prevalence was visualised via choropleth mapping using the ggplot2 and sf packages in R. Base map shapefiles for Thailand’s administrative boundaries were obtained from the Humanitarian Data Exchange (URL: https://data.humdata.org/dataset/cod-ab-tha), sourced from the Royal Thai Survey Department.”

**Results:**

The Results section contains valuable findings but needs streamlining to avoid redundancy and overly technical details. Keep focusing on key prevalence data, significant site differences, and species distribution; move excessive technical descriptions (bootstrap, SNPs) and interpretive statements to Supplementary or Discussion.

Thank you for your valuable suggestions.

We have removed the redundancy and overly technical details of categorised *Leptospira* clusters, SNPs, and bootstrap values (original Lines 260-279) from the Results and added them to Supplementary Document 1. The revision of the first paragraph of “*Leptospira* Pathogen clade detected in dogs and cats” is as follows:

Line 227-232	“The ML tree analysis (Supplementary Document 1) revealed that 39/56 PCR-positive dogs and cats were infected with *Leptospira* subclade P1 and P2. Five distinct clusters were identified (Fig 2): Interrogans (46%), Borgpetersenii (13%), Weilii (5%), Yasudae (15%) and undetermined species of P2 (21%). The P2 classifications, based on *rrs* sequence polymorphisms and the distribution of infection, are detailed in Supplementary Document 1. The distributions of each subclade are detailed in Supplementary Table 3.”

We will revise the other points with the details below.

**Line 218–224:**

-“looked and acted healthy” → replace with *“clinically healthy”*.

We have changed as you suggested. We also changed one more location found in Abstract as follows.

Line 203-204	“A total of 567 animals, comprising 303 dogs (53.4%) and 264 cats (46.6%), clinically healthy, were recruited into the study”

Line 43	“….in Southeast Asia, in clinically healthy owned and free-roaming dogs…”

- Condense province list — keep short here, refer to Fig. 1 or Supplementary Table.

We appreciate your suggestion. We agree that listing long province names can interrupt the reading flow. However, it is crucial to establish the regional context for these seven provinces. To balance readability with precision, we have moved the specific regional classifications to the legend of Fig 1, which is referenced in the text as follows.

Line 204-205	“… They originated from seven provinces in three regions of Thailand, with the infection frequencies shown in Fig. 1”

The legend of Fig 1(Line 145-151) “Fig 1. Geographic distribution of dog and cat Leptospira infections (Panel A) and median annual human leptospirosis incidence (Panel B) in seven provinces of Thailand: Nakhon Sawan, Nakhon Pathom, and Samut Sakhon (Central); Tak, Kanchanaburi, and Prachuap Khiri Khan (West); and Ranong (South). Infection frequencies in animals were identified using PCR and sequencing. Human incidence data (per 100,000 population) were reported by the Ministry of Public Health. Darker shades indicate higher prevalence/incidence. Base map shapefiles were obtained from The Humanitarian Data Exchange (sourced from the Royal Thai Survey Department).”

**Line 226–231 (Dogs):**

- Reporting is correct but repetitive. Instead of three sentences, summarize:
  *“Overall prevalence was 11.2% (34/303), with slightly higher infection frequencies in females (12.8%) vs males (8.8%), adults (11.9%) vs juveniles (6.1%), and free-roaming (11.8%) vs owned dogs (10.2%). None of these differences were statistically significant.”*

Considering these comments and the comments in Table 1 (Interpretation), together with comments for the dog and cat sections. We have revised in a summarised format as follows:

Line 210-216	“The overall *Leptospira* infection prevalence was 11.2% (34/303) in dogs and 8.3% (22/264) in cats. In both animal types, statistical analysis revealed no significant differences in prevalence by sex, age group, or ownership status (Table 1). Although age was not statistically significant, no infections were detected in juvenile cats, compared with an 8.8% rate in adults. Geographical location was a significant predictor of infection for dogs. Dogs in Tak and Prachuap Khiri Khan exhibited significantly higher infection risks compared to the Nakhon Pathom site (*p* < 0.05).”

- Keep only significant site-specific results highlighted (OR values).

We try to balance this comment with the comments on Tables 1 and 2 regarding “interpreting rare events ORs cautiously”. Therefore, we only informed the significant differences (with *p*-value) but did not specify (ORs) how strong the association is, which is shown in Line 210-216 (in the response to the comment above)

**Line 239–245 (Cats):**

- Similar issues can condense to one compact sentence.

We have revised as shown in the response to the comment above (Line 210-216).

- Clarify that infections were *only detected in adult cats*; highlight that juveniles were negative.

We have revised as shown in the response to the comment above (Line 210-216).

- Remove redundancy of “not statistically significant.”

We have revised as shown in the response to the comment above (Line 210-216).

**Line 246–258 (Human incidence):**

- Too interpretive for Results. Rephrase to present data only (incidence in Ranong vs Tak vs others).
- We have revised as follows.

Line 221-225	“National annual incidence data reported in those seven provinces (Supplementary document 2) indicate that Ranong had the highest human leptospirosis incidence (median 36.04 cases per 100,000 population), nearly 22-fold higher than Tak (1.65/100,000)(Fig 1). In contrast, our study found the highest leptospirosis prevalence in dogs and cats in Prachuap Khiri Khan (26.5%) and Tak (16.1%).”

- Sentence at 254–258 (“demonstrated that infections from dogs and cats can be transferred to humans”) is a *Discussion statement*. Suggest moving.

We have moved and revised in the Discussion section (see below, response to comments for Line 327-331, point 2).

**Line 287–294 (Geographic distribution):**

- Species distribution is useful, but wording is awkward. Example: “L. yasudae predominated in Prachuap Khiri Khan” → rephrase to *“L. yasudae (P1-2) was mainly detected in Prachuap Khiri Khan and, to a lesser extent, in Ranong.”*

We have revised the entire paragraph per your suggestion to ensure it flows smoothly with the previous paragraph and avoids redundancy. The revised text responds to comment of Lines 304-307 below.

- Consistently use correct name spelling (*Khan* not *Khun*).

We have corrected all consistently.

**Line 304–307 (Odds ratios):**

- This is important, keep. Just ensure OR results are placed in same paragraph with corresponding prevalence data (better flow).

We try to balance this comment with the comments on and 2 regarding “interpreting rare events ORs cautiously”. Therefore, we only informed the significant differences (with *p*-value) but did not specify (ORs) how strong the association is, as follows:

Line 241-251	“Geographically, P1-1 infections (*L. interorgans, L. borgpetersenii* and *L. weilii*) were most prevalent in animals from Tak (16.7% dogs vs 12.5% cats), followed by Ranong (6.5% vs 3.1%), Kanchanaburi (4.6% vs 4.8%), Prachuap Khiri Khan (3.6% vs 2.1%) and Nakhon Pathom (only 1.4% dogs) (Fig 3). Statistical analysis demonstrated that animals in Tak have a significantly higher risk of P1-1 infections than those in Nakhon Pathom (*p*-value = 0.005) (Table 2). *L. yasudae* (P1-2) was mainly detected in Prachuap Khiri Khan (5.5% vs 4.3%) and, to a lesser extent, in Ranong (0.8%, cats only). Animals from Prachuap Khiri Khan had a significantly higher risk of *L. yasudae* infections than those from Ranong (*p*-value = 0.010). The P2 cluster was most frequently found in Prachuap Khiri Khan (1.8% vs 6.4%), followed by Tak (4.2%, dogs only) and Ranong (0.9% vs 1.5%); the risk of P2 infection did not differ significantly among these three sites.”

**Discussion:**

- The Discussion is comprehensive and well referenced, however, it is too lengthy and descriptive, reading more like a literature review than a focused discussion.

Thank you for your valuable suggestions. We will revise as the details below.

- Needs focus on:
  1. Key findings (novelty and significance).
  2. Comparison with previous studies (concise, avoid long lists of countries/percentages).
  3. Public health and One Health implications.
  4. Limitations (already strong but could be condensed).
  5. Clear conclusion and recommendations.

Thank you for your valuable suggestions. We will revise as the details below.

**Line 312–319:**

- Replace “looked healthy” with *“clinically healthy”*.

We added “clinically” in front of the “healthy” as follows:

Line 264	“…the urine of clinically healthy (asymptomatic) dogs and cats”

- Condense comparisons — avoid listing prevalence for many countries; instead, group them (e.g., “higher in South America, moderate in Asia, lower in Europe”).

We have revised per your suggestion as follows:

Line 274-278	“The urinary shedding prevalence in this study was slightly higher than that reported in previous Thai studies [30-32, 43]. In dogs, our findings were comparable to those from Malaysia, the USA and several European nations [34, 38, 41, 45, 47], but remain lower than the high-burden regions of South Asia, South America and Iran, where the prevalences ranged between 10.6% and 97.4% [33, 37, 39, 42, 46, 48, 50, 53, 56]. …”

**Line 320–326:**

- The long list of high-prevalence countries (Sri Lanka, India, Brazil, Ecuador, etc.) is overwhelming. Summarize in 1–2 sentences instead of line-by-line reporting.

We have revised per your suggestion as follows:

Line 278-281	“… In cats, our prevalence fits within the broad international range of 0.3% to 12.9% reported in several countries of Asia, Europe, Latin America and the Caribbean [49, 58, 59, 61-67, 69], contrasting with the strikingly high prevalence reported in Taiwan (67.8%)[60]. …”

**Line 327–331:**

- Strong point: emphasize novelty of detecting *Leptospira* DNA in asymptomatic animals (urinary shedding).

We have revised per your suggestion as follows:

Line 283-285	“… The application of molecular methods to specifically identify pathogenic *Leptospira* DNA in clinically healthy carriers provides a crucial tool for monitoring transmission risks from these animals and informing targeted control strategies.”

- Suggest linking directly to risk of spillover to humans in Thailand.

Thank you for your valuable comment. We have moved the interpretation regarding potential transmission from dogs and cats to humans from the Results section to the first paragraph of the Discussion. This placement now directly follows the transmission risk assessment, creating a more logical flow and strengthening the narrative as follows:

Line 263-273	“This study reveals a critical public health risk: the shedding of pathogenic *Leptospira* in the urine of clinically healthy (asymptomatic) dogs and cats across central, western, and southern Thailand. Notably, 9.9% of these animals (11.2% dogs vs 8.3% cats) were identified as active shedders, posing a direct transmission risk to owners, other animals, and the environment. Regional analysis indicated that infection rates were lower in the central region compared to the western and southern regions. Given the national reports of human leptospirosis incidence, these findings suggest a potential for disease transmission from dogs and cats to humans within the same spatiotemporal context. However, companion animals are likely not the only sources of human infections in these provinces; other reservoirs (such as rodents and livestock) and environmental exposure via contaminated water or agricultural activities undoubtedly play significant roles in the transmission cycle.”

**Line 332–343:**

- Good identification of key pathogenic species (*L. interrogans, L. borgpetersenii, L. weilii*).

Thank you.

- Keep but tighten wording: avoid repeating country lists; instead highlight “consistent with reports across Asia, Europe, and Latin America.”

We have revised per your suggestion as follows:

Line 286-292	“Several *Leptospira* spp. within the Pathogen Clade have been reported in asymptomatic dogs and cats worldwide. Commonly identified pathogenic species include *L. interrogans*, *L. borgpetersenii*, *L. weilii*, *L. kirschneri*, *L. santarosai*, and *L. noguchii* [25, 31, 33, 34, 36, 37, 41, 42, 47-55, 57, 58, 60-64, 68]. Among these, *L. interrogans* predominates in both dogs and cats from Malaysia [34, 58] and is a primary species identified in North America & the Caribbean, South America, Europe, Africa Asia and Australia [25, 33, 36, 37, 42, 47, 52, 55, 58-60, 62, 63, 68]. …”

**Line 344–348:**

- Novelty claim: detection of *L. yasudae* (P1-2) in companion animals in Thailand. This is a *major strength* and should be emphasized earlier in the discussion as a key finding.

We have revised per your suggestion as follows:

Line 292-302

“… Consistent with these global trends, this study confirms that dogs and cats across Thailand carry these major pathogenic species. We identified *L. interrogans, L. borgpetersenii*, *and L. weillii* (which are the predominant causes of human leptospirosis in Thailand and neighbouring countries [77-84]) in dogs and cats during neutering procedures from western (Tak, Kanchanaburi, and Prachuap Khiri Khan), southern (Ranong), and central (Nakhon Pathom) regions.

Notably, beyond these established P1-1 pathogens, we report the first identification of *L. yasudae* (a species of undetermined virulence) in companion animals in western (Prachuap Khiri Khan) and southern (Ranong) Thailand. Furthermore, unspecified species of *Leptospira* within the P2 subclade (associated with mild diseases in humans [17]) was identified in southern (Ranong) and western (Tak and Prachuap Khiri Khan) regions.”

**Line 349–356:**

- Discussion of *L. interrogans* prevalence between dogs and cats is good.

Thank you.

- However, the statement “reason for host bias unknown” could be reframed more constructively (e.g., “may be due to differences in exposure ecology or sample size, but further studies are required”).

Thank you for this insight. We have reframed that sentence according to your suggestion. We have also revised the discussion regarding *L. interrogans* host pattern to better contrast our findings with the previous reports to create a more logical flow and strengthen the narrative as follows:

Line 303-311	“We detected *L. interrogans* in similar proportions in urine samples from dogs (3.6%) and cats (2.7%). In contrast, a previous report from northern Thailand (Nan) detected urinary shedding of *L. interrogans* in 6.9% of dogs, but not in cats [31]. Conversely, a study in Songkhla (southern Thailand) found a higher prevalence of *L. interrogans* in cats compared to dogs (0.5% of dogs’ vs 7.8% of cats’ blood PCR-positive) [43]. Nan and Songkhla were provinces where human leptospirosis was a significant problem, particularly during the wet season [21, 85]. The reasons for the variation in host infection rates across these areas remain unclear; further investigation is needed to determine if these host disparities result from local ecological exposure or sampling differences.”

**Line 357–364:**

- Novelty: first report of *L. borgpetersenii* in asymptomatic dogs/cats in Thailand. Keep this but make it more concise (currently there is too much background on cattle origins).

We have shortened the cattle background of *L. borgpetersenii* as follows:

Line 312-319	“*L. borgpetersenii*, a common cause of bovine leptospirosis, was first isolated from US slaughter cattle [86]. However, *L. borgpetersenii* infections have also been identified in dogs and cats. Notably, the number reported among cats was higher than that among dogs. For example, 7.1% of cats were infected on Okinawa Island, Japan [64], and 4.5% in southern Italy [61]. In contrast, 1.1% of dogs in Sri Lanka [33] and 0.5% in Germany [41] were infected. To our knowledge, the identification of *L. borgpetersenii* infections among asymptomatic dogs and cats has been reported for the first time in Thailand: in central (Nakhon Pathom) and southern (Ranong) regions.”

**Line 365–370:**

- Discussion of *L. weilii* is relevant but should be shortened; focus on Thai findings (low prevalence, dogs only).

We have revised the paragraph to focus more on our findings as follows:

Line 320-323	“*L. weilii* infects a broad host range, including livestock, canines and wildlife [31, 87, 88]. In this study, *L. weilii* infections were found in dogs with a low prevalence of 0.7% (Kanchanaburi and Ranong) and were not identified in cats. This contrasts with the higher canine infection rates in northern Thailand (3.4%) [31] and Sri Lanka (1.1%) [33].”

**Line 371–376:**

- Suggest highlighting as “the first detection of this species in companion animals globally/regionally.”

Thank you for the insightful suggestion. We have revised the paragraph to underscore the novelty of this first global report of *L. yasudae* in animals. Additionally, we have included details on the aseptic urine collection method to rule out environmental contamination explicitly. The revised text is as follows:

Line 324-329	“*L. yasudae* (conspecific with *L. dzianensis* [7]) was previously isolated solely from environmental samples, without documented reports of natural infection in humans or animals. Here, we provide the first description of *L. yasudae* infections in companion animals. Crucially, *L. yasudae* was detected in urine collected aseptically from the bladders, ruling out environmental contamination. These infections were identified predominantly in dogs and cats from Prachuap Khiri Khan and in a cat from Ranong.”

**Line 377–401 (P2 subclade):**

- Too technical for Discussion. SNP grouping details (Lines 380–384) and long lists of P2 species should be summarized and moved to Supplementary/Results.

We have moved the P2 classification details in the Supplementary Document 1.

- Instead, emphasize: *“P2 species, including L. wolffii and L. licerasiae, were detected, indicating potential emergence in animal reservoirs in Thailand.”*

Thank you for this insightful comment. We have revised the text to highlight that the detection of P2 species in our study indicates their potential emergence as animal reservoirs in Thailand. Furthermore, we have expanded this discussion to suggest a broader potential emergence across South and Southeast Asia. We believe this regional context is justified by recent reports of *L. wolffii* and *L. licerasiae* in countries such as Bangladesh, Iran, and Sri Lanka, suggesting that these species may be spreading or becoming more recognised across the continent. This broader perspective underscores the public health significance of our findings within the region. The revised paragraph is as follows:

Line 330-347	“Because of the limited diversity in the selected *rrs* sequences, *Leptospira* P2 could not be fully speciate. In stead, these undetermined species were classified into *L. wolffii*- or *L. licerasiae*-related groups (Supplementary Document 1). Members of P2 have been primarily isolated from environmental samples worldwide, including Mayotte, Puerto Rico, Japan, and Thailand [12, 82, 89-91]. The potentially pathogenic *L. wolffii* was first documented in a patient from northeastern Thailand and subsequently identified in local rodents [17, 92]. Recently, *L. wolffii* infections have been reported in other regions, including Iran and north-central Bangladesh, where it has reportedly replaced *L. interrogans* as a primary species [71, 93-95]. In this study, we identified urinary shedding of the *L. wolffii*-related group in six animals from Ranong and Prachuap Khiri Khan. Similarly, *L. licerasiae* (associated with mild symptomatic infections) was first described in rats and humans in (or returning from) South America [96, 97] and has since been detected in Australian swine and leptospirosis-vaccinated dogs in Sri Lanka [48, 98]. We identified two animals carrying the *L. licerasiae*-related group in Tak and Ranong. These findings underscore the potential emergence of *L. wolffii*- and *L. licerasiae*-related infections in animal reservoirs in Thailand, as well as across South and Southeast Asia. Although the precise species within the P2 subclade infecting these dogs and cats remains undetermined, the broad geographical distribution of these reservoirs warrants further attention regarding their implications for animal and public health.”

**Line 402–421 (Limitations):**

- Strong section but wordy, condense to 3–4 main points:
  1. Unequal sample size across sites.
  2. Geographic coverage is incomplete.
  3. Not all positives are sequenced due to DNA yield.
  4. Partial 16S rRNA sequencing limited in species resolution, especially P2.

We appreciate the guidance to streamline this section. Following your suggestion, we have consolidated the limitations into three main thematic points:

1. Study Design and Scope: Addressing unequal sample sizes and geographic coverage.
2. Sensitivity and Underestimation: Addressing the biological and logistical factors that may lead to false negatives (as requested by Reviewer #2).
3. Molecular and Technical Constraints: Addressing DNA yield, primer competition, and the resolution limits of the *rrs* gene, particularly for the P2 subclade.

The revised paragraphs are as follows:

Line 348-371	“This study has several limitations to consider. First, the geographic scope and sample distribution were restricted. Sampling was limited to central, western, and southern Thailand, and sample sizes were unevenly distributed across sites (median = 41.5; range = 12–238). This imbalance may have reduced the statistical power to detect infections in sites with low recruitment, suggesting that these results may not fully represent the epidemiological situation in the northern or eastern regions of the country. Second, the reported prevalence likely underestimates the true infection rate. As our study focused on urinary shedding (leptospiruria) to identify chronic carriers, we did not perform blood sample investigation; consequently, animals in the acute phase of infection (where the pathogen is present in the blood but not yet shed in the urine) were not captured. Furthermore, because *Leptospira* are shed intermittently [25, 42], a single-point urine collection may have yielded false-negative results for some carriers. Other factors also contribute to this underestimation, including potential false PCR negatives due to antibiotic administration before urine collection (a factor we could not fully control, though our neutering protocol did not use antibiotics). Third, logistical constraints impacted molecular identification. Due to limited field resources, samples were maintained at 2°C–6°C without pH neutralisation. The resulting exposure to potentially acidic urine, particularly in samples requiring prolonged transport (>24 hours) from distant sites, may have caused DNA degradation and reduced diagnostic sensitivity. Additionally, species identification was limited by low DNA yields and potential primer competition, leading to ambiguous Sanger sequencing reads. While the conserved 443 bp *rrs* gene fragment is a rapid method for identifying major P1 subclade species, it cannot accurately distinguish between members of the P2 subclade due to low genetic diversity in this region [9]. Future studies could achieve higher resolution for P2 species by targeting a more diverse set of markers, such as *lipL32* and *secY* [89], or by employing next-generation deep sequencing.”

**Conclusion**

**Line 422–434:**

- Well-structured but could be more concise.

Thank you. We have condensed the paragraph per your suggestions, shown in the next comments’ response.

- Suggest rephrasing final sentence: *“Our findings highlight the role of dogs and cats as reservoirs of pathogenic Leptospira spp., emphasizing the need for expanded surveillance, broader-spectrum vaccines, and public awareness campaigns to mitigate zoonotic risk.”*

We have added the sentence and condensed the inclusion paragraph as follows:

Line 372-379	“In conclusion, three classical *Leptospira* species (*L. interrogans*, *L. weilii*, and *L. borgpetersenii*), responsible for human leptospirosis, were predominantly identified in dogs and cats in western and southern Thailand. Additionally, species potentially causing mild leptospirosis (*L. wolffii*- and *L. licerasiae-*related groups) and those with unknown infectivity (*L. yasudae*) were also detected. Our findings highlight the role of dogs and cats as reservoirs of pathogenic *Leptospira* spp., emphasising the need for expanded surveillance, broader-spectrum vaccines to reduce urinary shedding, and public awareness campaigns to mitigate zoonotic risk.”

**Figure 1**

- Province names are clear but ensure consistency: “Prachuap Khiri Khan” is misspelled as “Prachuap Khiri Khun” in the manuscript text.

Thank you. We have checked the consistency and corrected it.

- Infection data in parentheses (e.g., “9/56”) is useful — keep, but standardize formatting with percentages (e.g., “16.1% (9/56)”).

Thank you. We have corrected them (0 become to 0%) for Samut Sakhon and Nakhon Sawan

**Interpretation:**

- *The caption is long and somewhat repetitive. Suggest shortening:
  “Fig 1. Geographic distribution of dog and cat Leptospira infections (Panel A) and median annual human leptospirosis incidence (Panel B) in seven provinces of Thailand. Infection frequencies in animals were identified using PCR and sequencing. Human incidence data (per 100,000 population) were reported by the Ministry of Public Health. Darker shades indicate higher prevalence/incidence.”*

Thank you for providing the improved caption text, which we have adopted. However, as per your previous suggestion to remove the detailed list of province names from the main text, we have added these names to the Fig 1 caption. We believe this is essential so that readers can identify the specific study sites and regions without cluttering the Results section. The caption now reads:

“Fig 1. Geographic distribution of dog and cat Leptospira infections (Panel A) and median annual human leptospirosis incidence (Panel B) in seven provinces of Thailand: Nakhon Sawan, Nakhon Pathom, and Samut Sakhon (Central); Tak, Kanchanaburi, and Prachuap Khiri Khan (West); and Ranong (South). Infection frequencies in animals were identified using PCR and sequencing. Human incidence data (per 100,000 population) were reported by the Ministry of Public Health. Darker shades indicate higher prevalence/incidence. Base map shapefiles were obtained from The Humanitarian Data Exchange (sourced from the Royal Thai Survey Department).”

**Table 1**

- Column headings (“infected (%)”, “subtotal”) could be standardized to: *“n infected (%)”* and *“Total (n)”* for clarity.

We have changed as you suggested.

- For provinces with very wide confidence intervals (e.g., OR = 17.290, 95% CI: 4.373–133.623), it would be helpful to note the limitation due to small sample size.

We have added “#” to denote the wide confidence intervals as you suggested and added the footnote below Table 1. as follows:

“^#^The large interval of 95% confidence intervals suggests the limitation due to the small sample size.”

- The footnotes mention Fisher’s exact test but the main text references normal approximation; clarify which was applied in each case.

We have clarified these inconsistencies in the method section and the footnote below Table 1 as follows:

Line 189-194 (Method)	“…To determine the strength of associations between prevalence and exposure factors, Odds Ratios (ORs) and 95% confidence intervals (CIs) were calculated using the 'epitools' package for R [76]. Small-sample adjustments were applied to both OR estimates and CIs (using a normal approximation), and p-values were derived using Fisher's exact test. Statistical significance was defined as a *p*-value < 0.05 and a 95% CI that did not include 1. …”

Table 1 footnote	“*Notify the statistically significant:*p*-value less than 0.05, which was calculated using Fisher's exact method, and the entire range of the 95% confidence interval is more than 1 or less than 1, which was calculated using normal approximation with the small sample adjustment.”

- Shade significant results (e.g., *p* < 0.05) in bold font to help readers quickly identify important findings.

We thank the reviewer for this suggestion. We apologise for any formatting issues in the previous version. We have double-checked all tables to ensure that statistically significant results (p < 0.05) are clearly marked in bold.

**Interpretation**

- The table itself shows most demographic variables are not significant predictors, except site-specific differences in dogs. This should be emphasized in the Results section instead of repeating “not significant.”

We thank the reviewer for this constructive suggestion to improve the narrative flow. We agree that listing specific prevalence rates for non-significant variables created unnecessary redundancy.

Accordingly, we have streamlined the Results section by grouping the non-significant demographic variables (sex, age, ownership) for both dogs and cats into a single statement. This revision minimises the emphasis on non-predictive factors. It allows the significant geographical predictors in dogs, as well as the notable absence of infection in juvenile cats, to stand out clearly, as shown in the response to the comment of Line 226–231 (Dogs) above.

**Table 2**:

- Wide CIs due to small subgroups; note limitations. Interpret rare events ORs cautiously.

Thank you. We added the limitation in the footnote, consistent with Table 1. We have revised the finding without specifying the OR as follows:

Line 244-246	“… Statistical analysis demonstrated that animals in Tak have a significantly higher risk of P1-1 infections than those in Nakhon Pathom (*p*-value = 0.005) (Table 2). …”

Line 247-248	“… Animals from Prachuap Khiri Khan had a significantly higher risk of *L. yasudae* infections than those from Ranong (*p*-value = 0.010). …”

- Emphasize novelty of *L. yasudae* detection and geographic risk differences (Tak, Prachuap Khiri Khan).

Thank you. We have done it as a response to the comment above.
