# Supplementary material for: Pathogenic Leptospira species identified in dogs and cats during neutering in Thailand
Source: PLoS Negl Trop Dis. 2026 Feb 4;20(2):e0013421. doi: 10.1371/journal.pntd.0013421 (PMC12871963; doi:10.1371/journal.pntd.0013421)
Supplement: Attachment — Submitted filename: Reviewer Comments PNTD-D-25-01286.docx [file pntd.0013421.s006.docx]

**Reviewer Comments**

**General:**

This study provides valuable One Health data on asymptomatic *Leptospira* infections in dogs and cats, with the novel detection of *L. yasudae* and P2 clade species adding to its significance. The methodology is sound, and the results are clearly supported by the data. The main areas for improvement are reducing overly descriptive sections in the Introduction and Discussion and moving some technical details from the Methods/Results to the Supplementary Material for clarity. Overall, the manuscript is strong and requires only minor revisions before acceptance.

**Abstract**

**Line 26:** Revise phrasing → *“Pathogenic species of the genus Leptospira cause an underdiagnosed zoonosis…”* for scientific accuracy.

**Line 27–28:** Reword → *“Animal reservoirs often remain asymptomatic yet shed the bacteria in urine…”* to improve clarity.

**Line 30:** Replace *“leading to unawareness of infections”* with *“resulting in unrecognized infections”*.

**Line 32–33:** Consider stating the knowledge gap in Thailand to highlight novelty.

**Line 34:** Simplify → *“We investigated the prevalence of Leptospira infection in cats and dogs in Thailand.”*

**Line 36–37:** Replace *“screened for Leptospira’s DNA”* with *“screened for Leptospira DNA by PCR targeting the rrs gene.”*

**Line 37–41:** Present prevalence more clearly: first overall (56/567, 9.9%), then breakdown by dogs (11.2%) and cats (8.3%).

**Line 39–41:** Emphasize novelty → *“This represents the first detection of L. yasudae in companion animals in Thailand.”*

**Line 42–45:** Replace casual phrase *“probably living under the same roof as us”* with a more formal version: *“These companion animals, often living in close contact with humans, may contribute to daily transmission risks.”*

**Line 46–47:** Strengthen conclusion → *“Enhanced surveillance and vaccination of dogs and cats, combined with public awareness campaigns, may reduce human infection risks.”*

**Line 48:** Keywords are fine; consider adding *“molecular epidemiology”* for better indexing.

**Introduction:**

**Overall:**

- Streamline taxonomy and prevalence details.
- Ensure statistics are current and contextualized.
- Reorganize flow: global context → Thailand data → pets as reservoirs → knowledge gap → study aim.

**Line 68–74:**

- Revise wording for clarity (*“The genus Leptospira comprises Gram-negative spirochetes…”*).
- Merge sentences on carriers and transmission to reduce repetition.

**Line 75–85:**

- Taxonomy section is too detailed; condense. Keep only clinically relevant species (e.g., *L. interrogans, L. borgpetersenii, L. weilii, L. yasudae*).
- Avoid long lists of rarely reported species in the Introduction.

**Line 86–95:**

- Update global burden statistics (current WHO/GBD).
- Simplify risk factors sentences; split into two shorter sentences for readability.

**Line 96–105:**

- Strengthen rationale for companion animals as reservoirs.
- Reword casual phrasing (*“bring pathogens from outside into houses” → “may introduce pathogens into households”*).
- Condense pet population statistics (Line 102–103). Suggest including “Athapattu T, Fernando R, Abayawansha R, Fernando P, Fuward M, Samarakoon N, Koizumi N, Gamage C. Carrier Status of *Leptospira* spp. in Healthy Companion Dogs in Sri Lanka. Vector Borne Zoonotic Dis. 2022 Feb;22(2):93-100. doi: 10.1089/vbz.2021.0065.”

**Line 106–113:**

- Global prevalence numbers (97% dogs, 68% cats) seem like an inflated clarify method (PCR vs serology).
- Line 107 citation of references seems to be incorrect, isn’t it 30, 70? recheck.

**Line 114–117:**

- Knowledge gap statement (“scarce in Thailand”) should be moved earlier for emphasis.
- Objective sentences should be tightened for clarity and impact.

**Methods:**

Methods are rigorous and reproducible but too detailed in technical parameters. Streamlining main text while moving exhaustive PCR/phylogenetic details to Supplementary would improve readability and fit *PLOS NTDs* style.

**Ethics Statement**

**Line 120–127**

- Well written and includes both IACUC and Biosafety approvals.
- Clarify whether animal welfare guidelines (e.g., ARRIVE, OIE, or national standards) were followed. PLOS journals often require explicit mention of compliance with international standards.
- Consider condensing slightly: consent procedures could be summarized in fewer sentences.

**Sample Size Calculation**

**Line 128–133**

- Appropriate approach using ProMESA software.
- Relative error of 2% seems unusually strict and may inflate sample size requirements. Clarify why this threshold was chosen.

**Study Population**

**Line 134–141**

- Wording “dogs and cats that look healthy” → should be *“clinically healthy dogs and cats”*.
- Ownership categories should be standardized (e.g., “owned” vs. “free-roaming/unowned”).
- Suggestion: Add numbers by group (owned vs free-roaming) if available. This will be useful for interpretation later.

**Line 138–140:**

- Province listing is correct but long; consider replacing with *“…across seven provinces in central, western, and southern Thailand”* and keep full details in Fig. 1 or Supplementary Table.

**Urine Collection and DNA Extraction**

**Line 154–165**

- Volume range (dogs: 1–370 mL) is extremely wide; clarify whether very large outliers are from larger animals or pooled samples.
- Specify how long samples were stored before processing. This has effect on DNA degradation.

**PCR and Sequencing**

**Line 166–187**

- Methods are detailed, but overly technical for an Introduction-to-Methods section. PCR primer sequences and thermocycler details are usually moved to Supplementary Methods.
- The primer names could be formatted in italics or in Supplementary Data for clarity.

**Line 187:** “would be sent to Bionics” → rephrase to *“were sent to Bionics (South Korea) for Sanger sequencing.”*

**Phylogenetic Analysis**

**Line 188–207**

- Description is too detailed for main Methods. Readers need to know:
  - Alignment length, Model used and Software version.
- Details on gamma distribution parameters (+G = 0.1284) and invariant sites (42.89%) can be shortened or placed in Supplementary Material.
- Condense to ~5 sentences in main text; move parameter-heavy details to Supplementary Table.

**Statistical Analysis**

**Line 208–215**

- Clear and appropriate (ORs with small sample adjustment, 95% CIs).
- Mention significance threshold (e.g., *p* < 0.05).
- Clarify whether multiple testing corrections were applied.
- Good to note mapping was done using Excel but could also specify GIS software if used (Excel maps may raise questions about precision).

**Results:**

The Results section contains valuable findings but needs streamlining to avoid redundancy and overly technical details. Keep focusing on key prevalence data, significant site differences, and species distribution; move excessive technical descriptions (bootstrap, SNPs) and interpretive statements to Supplementary or Discussion.

**Line 218–224:**

- “looked and acted healthy” → replace with *“clinically healthy”*.
- Condense province list — keep short here, refer to Fig. 1 or Supplementary Table.

**Line 226–231 (Dogs):**

- Reporting is correct but repetitive. Instead of three sentences, summarize:
  *“Overall prevalence was 11.2% (34/303), with slightly higher infection frequencies in females (12.8%) vs males (8.8%), adults (11.9%) vs juveniles (6.1%), and free-roaming (11.8%) vs owned dogs (10.2%). None of these differences were statistically significant.”*
- Keep only significant site-specific results highlighted (OR values).

**Line 239–245 (Cats):**

- Similar issues can condense to one compact sentence.
- Clarify that infections were *only detected in adult cats*; highlight that juveniles were negative.
- Remove redundancy of “not statistically significant.”

**Line 246–258 (Human incidence):**

- Too interpretive for Results. Rephrase to present data only (incidence in Ranong vs Tak vs others).
- Sentence at 254–258 (“demonstrated that infections from dogs and cats can be transferred to humans”) is a *Discussion statement*. Suggest moving.

**Line 287–294 (Geographic distribution):**

- Species distribution is useful, but wording is awkward. Example: “L. yasudae predominated in Prachuap Khiri Khan” → rephrase to *“L. yasudae (P1-2) was mainly detected in Prachuap Khiri Khan and, to a lesser extent, in Ranong.”*
- Consistently use correct name spelling (*Khan* not *Khun*).

**Line 304–307 (Odds ratios):**

- This is important, keep. Just ensure OR results are placed in same paragraph with corresponding prevalence data (better flow).

**Discussion:**

- The Discussion is comprehensive and well referenced, however, it is too lengthy and descriptive, reading more like a literature review than a focused discussion.
- Needs focus on:
  1. Key findings (novelty and significance).
  2. Comparison with previous studies (concise, avoid long lists of countries/percentages).
  3. Public health and One Health implications.
  4. Limitations (already strong but could be condensed).
  5. Clear conclusion and recommendations.

**Line 312–319:**

- Replace “looked healthy” with *“clinically healthy”*.
- Condense comparisons — avoid listing prevalence for many countries; instead, group them (e.g., “higher in South America, moderate in Asia, lower in Europe”).

**Line 320–326:**

- The long list of high-prevalence countries (Sri Lanka, India, Brazil, Ecuador, etc.) is overwhelming. Summarize in 1–2 sentences instead of line-by-line reporting.

**Line 327–331:**

- Strong point: emphasize novelty of detecting *Leptospira* DNA in asymptomatic animals (urinary shedding).
- Suggest linking directly to risk of spillover to humans in Thailand.

**Line 332–343:**

- Good identification of key pathogenic species (*L. interrogans, L. borgpetersenii, L. weilii*).
- Keep but tighten wording: avoid repeating country lists; instead highlight “consistent with reports across Asia, Europe, and Latin America.”

**Line 344–348:**

- Novelty claim: detection of *L. yasudae* (P1-2) in companion animals in Thailand. This is a *major strength* and should be emphasized earlier in the discussion as a key finding.

**Line 349–356:**

- Discussion of *L. interrogans* prevalence between dogs and cats is good.
- However, the statement “reason for host bias unknown” could be reframed more constructively (e.g., “may be due to differences in exposure ecology or sample size, but further studies are required”).

**Line 357–364:**

- Novelty: first report of *L. borgpetersenii* in asymptomatic dogs/cats in Thailand. Keep this but make it more concise (currently there is too much background on cattle origins).

**Line 365–370:**

- Discussion of *L. weilii* is relevant but should be shortened; focus on Thai findings (low prevalence, dogs only).

**Line 371–376:**

- Suggest highlighting as “the first detection of this species in companion animals globally/regionally.”

**Line 377–401 (P2 subclade):**

- Too technical for Discussion. SNP grouping details (Lines 380–384) and long lists of P2 species should be summarized and moved to Supplementary/Results.
- Instead, emphasize: *“P2 species, including L. wolffii and L. licerasiae, were detected, indicating potential emergence in animal reservoirs in Thailand.”*

**Line 402–421 (Limitations):**

- Strong section but wordy, condense to 3–4 main points:
  1. Unequal sample size across sites.
  2. Geographic coverage is incomplete.
  3. Not all positives are sequenced due to DNA yield.
  4. Partial 16S rRNA sequencing limited in species resolution, especially P2.

**Conclusion**

**Line 422–434:**

- Well-structured but could be more concise.
- Suggest rephrasing final sentence: *“Our findings highlight the role of dogs and cats as reservoirs of pathogenic Leptospira spp., emphasizing the need for expanded surveillance, broader-spectrum vaccines, and public awareness campaigns to mitigate zoonotic risk.”*

**Figure 1**

- Province names are clear but ensure consistency: “Prachuap Khiri Khan” is misspelled as “Prachuap Khiri Khun” in the manuscript text.
- Infection data in parentheses (e.g., “9/56”) is useful — keep, but standardize formatting with percentages (e.g., “16.1% (9/56)”).

**Interpretation:**

- *The caption is long and somewhat repetitive. Suggest shortening:
  “Fig 1. Geographic distribution of dog and cat Leptospira infections (Panel A) and median annual human leptospirosis incidence (Panel B) in seven provinces of Thailand. Infection frequencies in animals were identified using PCR and sequencing. Human incidence data (per 100,000 population) were reported by the Ministry of Public Health. Darker shades indicate higher prevalence/incidence.”*

**Table 1**

- Column headings (“infected (%)”, “subtotal”) could be standardized to: *“n infected (%)”* and *“Total (n)”* for clarity.
- For provinces with very wide confidence intervals (e.g., OR = 17.290, 95% CI: 4.373–133.623), it would be helpful to note the limitation due to small sample size.
- The footnotes mention Fisher’s exact test but the main text references normal approximation; clarify which was applied in each case.
- Shade significant results (e.g., *p* < 0.05) in bold font to help readers quickly identify important findings.

**Interpretation**

- The table itself shows most demographic variables are not significant predictors, except site-specific differences in dogs. This should be emphasized in the Results section instead of repeating “not significant.”

**Table 2**:

- Wide CIs due to small subgroups; note limitations. Interpret rare events ORs cautiously.
- Emphasize novelty of *L. yasudae* detection and geographic risk differences (Tak, Prachuap Khiri Khan).
